# AMPK regulated metabolic programing: oncogenic or growth suppressive? Evolving lessons from genetic and pharmacologic studies

**DOI:** 10.1186/2049-3002-2-S1-O23

**Published:** 2014-05-28

**Authors:** Xiaona Liu, Rishi Raj Chhipa, Lionel ML Chow, Matthew Wortman, Christopher McPherson, Ady Kendler, Biplab Dasgupta

**Affiliations:** 1 Department of Oncology, Cincinnati Children’s Hospital Medical Center, Cincinnati, OH, USA; 2 Department of Internal Medicine, University of Cincinnati, Cincinnati, OH, USA; 3 Department of Neurosurgery, Brain Tumor Center, University of Cincinnati Neuroscience Institute and Mayfield Clinic, Cincinnati, OH, USA; 4 Department of Pathology and Laboratory Medicine, University of Cincinnati, Cincinnati, OH, USA

## Background

Cancer cells reprogram their metabolism for optimal growth and survival. Identifying the genes and their functions crucial for cancer metabolic reprograming might have therapeutic implications. The multifunctional kinase AMPK is an evolutionarily conserved energy sensor that plays an important role in cell proliferation, growth and survival. It remains unclear whether AMPK functions as a tumor suppressor or a contextual oncogene. This is because while on one hand active AMPK inhibits mTOR and lipogenesis - two crucial arms of cancer growth, AMPK also ensures viability during metabolic stress. Many studies have shown that AMPK activation by two indirect AMPK agonists AICAR and metformin (now in many cancer clinical trials) reduces cancer cell proliferation - an effect that is rescued by an AMPK inhibitor Compound C in some studies. We used genetic models to scrutinize the specificity of these reagents and examine whether AMPK is a growth suppressor in glioblastoma.

## Materials and methods

We used silencing RNA against AMPK in established GBM cell lines and examined viability in the presence or absence of the reagents to define their mechanisms of action.

## Results

Surprisingly, we found that compared to normal tissue, AMPK is constitutively active in both human and mouse gliomas *in vivo*. We found that both AMPK agonists inhibited proliferation, but through discrete AMPK-independent mechanisms and both reagents reduced GBM growth *in vivo* independent of AMPK. Importantly, A769662, a new and direct AMPK activator had no effect on GBM cell proliferation. Metformin directly inhibited mTOR by enhancing PRAS40 association with RAPTOR, while AICAR blocked cell cycle through proteasomal degradation of the G2M phosphatase cdc25c. In another surprise, Compound C itself proved to be a potent anti-GBM agent; however, its cell killing effects were pleiotropic and also independent of AMPK. We next examined if AMPK is required for viability of freshly established patient-derived GBM cells and found that many of these cells lose viability *in vitro* when AMPK was silenced.

## Conclusions

Our results suggest that AICAR, metformin and Compound C are all AMPK-independent anti-proliferative agents. Importantly, physiologically active AMPK in GBM could be a growth promoter rather than a growth suppressor *in vivo*. We are taking multiple genetic approaches to determine this in mouse models of human GBM.

